# Optically pumped magnetoencephalography in epilepsy

**DOI:** 10.1002/acn3.50995

**Published:** 2020-02-29

**Authors:** Umesh Vivekananda, Stephanie Mellor, Tim M. Tierney, Niall Holmes, Elena Boto, James Leggett, Gillian Roberts, Ryan M. Hill, Vladimir Litvak, Matthew J. Brookes, Richard Bowtell, Gareth R. Barnes, Matthew C. Walker

**Affiliations:** ^1^ Wellcome Centre for Human Neuroimaging UCL Queen Square London WC1N 3AR United Kingdom; ^2^ Department of Clinical and Experimental Epilepsy UCL, Queen Square Institute of Neurology London WC1N 3BG United Kingdom; ^3^ Sir Peter Mansfield Imaging Centre School of Physics and Astronomy University of Nottingham Nottingham NG7 2RD United Kingdom

## Abstract

We demonstrate the first use of Optically Pumped Magnetoencephalography (OP‐MEG) in an epilepsy patient with unrestricted head movement. Current clinical MEG uses a traditional SQUID system, where sensors are cryogenically cooled and housed in a helmet in which the patient’s head is fixed. Here, we use a different type of sensor (OPM), which operates at room temperature and can be placed directly on the patient’s scalp, permitting free head movement. We performed OP‐MEG recording in a patient with refractory focal epilepsy. OP‐MEG‐identified analogous interictal activity to scalp EEG, and source localized this activity to an appropriate brain region.

## Introduction

Magnetoencephalography (MEG) is a noninvasive brain imaging technique that gives a unique window into whole brain function. In the field of epilepsy, MEG has been the source of much interest due to significant technical advantages it possesses over the ‘gold standard’ noninvasive technique for functional recording; scalp electroencephalography (EEG). Unlike EEG, the MEG signal is not affected by the smearing effects of skull and scalp and has better immunity to muscle artifact, both of which enable a greater ability to detect epileptic activity.^1^ MEG has been shown to provide useful additional information to EEG, which significantly contributes to patient selection, focus localization, and long‐term seizure freedom after epilepsy surgery.^2^, ^3^, ^4^ However, use of MEG in epilepsy thus far has been limited to the presurgical evaluation of patients with drug‐refractory epilepsies. This is because current clinical MEG uses an array of cryogenically cooled sensors termed superconducting quantum interference devices or SQUIDs, placed around the head,^5^, ^6^ making MEG systems cumbersome and restrictive as sensor positions are fixed in a limited selection of helmet sizes. Consequently, any motion of the head relative to the sensors, for example, during a seizure, affects MEG signal quality in spite of movement compensation algorithms applied to it.^7^ This means that MEG recording sessions are usually brief (1–2 h) when compared with EEG telemetry that can last several days.

Here, we describe the first use of an optically pumped (OP) MEG system^8^ in a case of medically refractory focal epilepsy. OP‐MEG utilizes novel quantum sensors (optically pumped magnetometers or OPMs) that do not rely on superconducting technology, but on the transmission of laser light through a vapor of spin‐polarized rubidium atoms. The use of OPMs in epilepsy has already been demonstrated in rodent models.^9^ Crucially, OPM sensors can be worn directly on the head, allowing the subject to move within a magnetically shielded environment while being scanned. A recent OPM study showed sensory motor signals robust to subject movement of 20 cm.^8^ In addition, as the magnetically sensitive volume within the OPM sits just 6 mm from the scalp surface by comparison to roughly 3–4 cm in cryogenic MEG, the magnetic field strengths measured due to cortical sources are typically four times greater in adults^10^, ^11^ as magnetic field strength decreases with distance. The white noise floor of the most recent OPMs is comparable to that SQUIDs (~10fT/sqrt(Hz)).^8^

In this study, we demonstrate, for the first time in humans, that interictal epileptiform activity can be recorded using OPMs. The localization of this activity agrees with that of other modalities. OP‐MEG therefore promises a combination of high spatiotemporal accuracy during brain recording (with minimal effects of motion or muscle artifact) and, practicality, with subjects able to move naturally during recordings.

## Method

Our test case was a 47‐year‐old woman who had Hemophilus meningitis associated with status epilepticus aged 18 months. After a period of seizure freedom, she developed focal seizures with loss of awareness, refractory to a number of antiepileptic drugs, and she is currently experiencing up to 10 seizures a day. Repeated diagnostic EEG demonstrated epileptic spikes, polyspikes, and spike and wave patterns indicative of a right posterior quadrant focus for the epilepsy. MRI demonstrated right‐sided parieto‐occipital damage of cortex and white matter, indicative of a previous ischemic insult.

**Figure 1:**
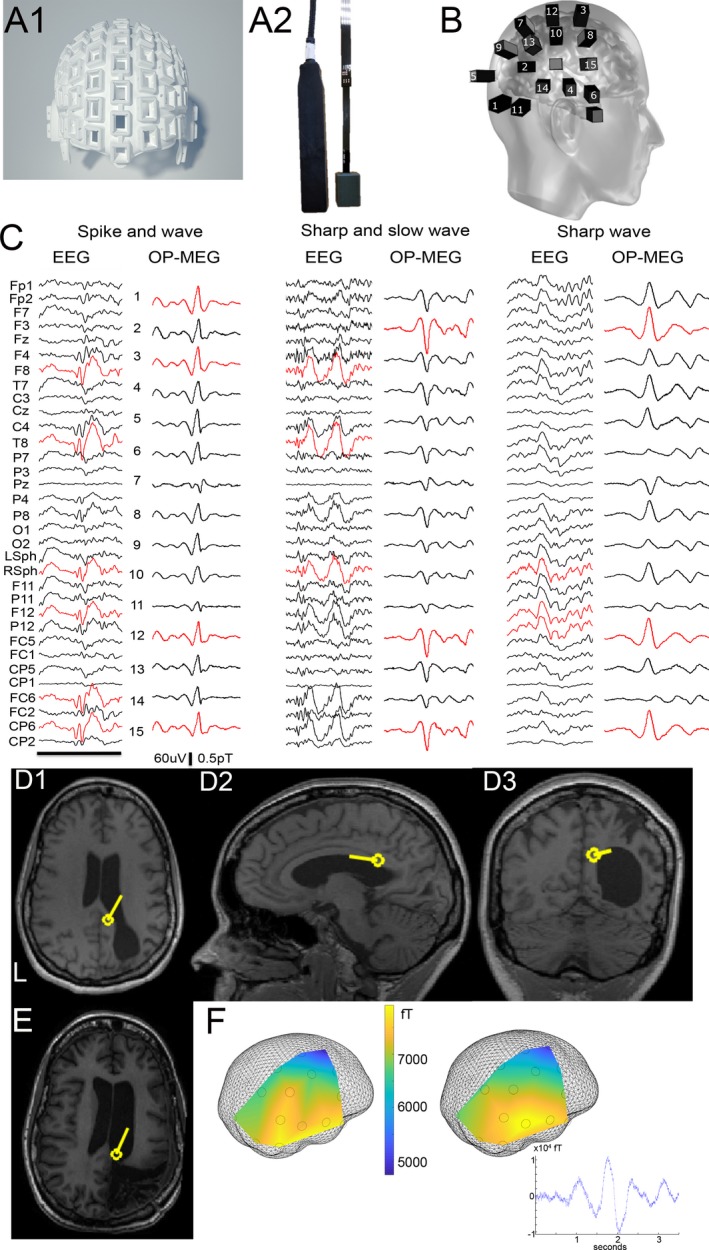
Use of OP‐MEG in epilepsy. (A1) Rendered image of 3D scanner‐cast. (A2) First‐generation (left) and second‐generation (right) OPM sensor. (B) Schematic of position of OPM sensors on the scalp for second recording session. (C) Example traces of typical epileptiform activity from scalp EEG and OP‐MEG including spike and wave, sharp and slow wave, and sharp wave. Red denotes channels with highest‐amplitude spikes. EEG labeled with average reference montage; Black bar = 1 second. (D) Source reconstruction of average interictal spike activity using OP‐MEG; D1 coronal, D2 sagittal, and D3 axial using dipole fit. (E) Postoperative MRI demonstrating likely removal of same average MEG dipole. (F) Field map (measured left and modeled right) corresponding to the epileptic spike with sensor positions shown in relation to the inner skull mesh. Inset shows example average OP‐MEG sharp wave used for reconstruction.

Prior ethical approval for this study was granted by The Medicines and Healthcare products Regulatory Agency. Informed consent was obtained from the patient to participate in the study. Band‐pass filtering between 1 and 70Hz was applied to the EEG and OP‐MEG data. Prolonged EEG monitoring (5 days) was performed with a MedTronic system with a 21‐channel setup using the international 10‐20 electrode system and average reference montage. The patient subsequently underwent two separate recording sessions using OP‐MEG, each lasting 30 min. For both recordings, we used remote reference sensors placed near the patient to record, and eventually regress out, environmental magnetic noise. The first session involved the patient lying on a bed with her head resting on a bespoke plinth, which housed eight first‐generation OPMs (Figure 1A2, left) for recording brain activity and six sensors were used for reference measurement. The second session was performed with the subject wearing a bespoke 3D‐printed scanner cast (Figure 1A1) to hold 15 second‐generation OPMs (Figure 1A2, right). The scanner‐cast design was informed by a previously acquired anatomical 3 T MRI scan.^12^ These casts provide a rigid sensor mount with respect to the individual anatomy and ensure that any movement artifacts are common across sensors. The positions of the sensors in relation to the brain could be ascertained directly from the digital scanner cast design (Figure 1B). Source reconstruction of an average of interictal spikes (manually identified by an experienced neurophysiologist, MW) was performed using dipole fitting within Fieldtrip software (http://fieldtriptoolbox.org) (Figure 1D–F). We used the rising phase to maximal peak of spike for reconstruction. Both recordings were performed within a magnetically shielded room and with the subject sitting between two biplanar coils separated by 1.5 meters, which were used to remove remnant static magnetic fields.^13^ This allowed the patient to move her head naturally while reclining in a chair.

## Results

During EEG telemetry the patient exhibited frequent interictal activity including sharp waves, spikes and polyspikes, on average 30/hour (examples in Figure 1C). These were maximal in channels T8 and Right Sphenoidal. She also had four seizures heralded by right fronto‐centro‐temporal spikes and subsequent generalized attenuation of EEG activity. We were able to identify the same interictal patterns, on 23 spikes/30 min during the first OP‐MEG recording session (Figure S1), and 19 spikes/30 min during the second session (Figure 1C). The patient suffered no seizures during OP‐MEG recording. Using spikes from the second session, where sensor position in relation to the brain could be defined, we localized the epileptiform activity to the previously EEG‐identified abnormal right posterior quadrant, assumed to be the epileptogenic focus (Figure 1D). She since underwent resective surgery (planned prior to the OPM recordings) of this region with a reduction in her seizure frequency (Figure 1E).

## Discussion

We have demonstrated the first use of OP‐MEG in an epilepsy patient with unrestricted head movement. OP‐MEG detected forms of abnormal interictal activity seen on clinical EEG (spikes, sharp and slow waves, polyspikes, and spike and waves) demonstrating similar morphology, and produced consistent localization of spike activity. One limitation of this study has been that EEG and OP‐MEG were not acquired at the same time. However, this study is an important proof‐of‐concept step in the use of OP‐MEG for epilepsy. Another limitation is that the OP‐MEG sensors were concentrated around the region of interest when recording; that is, right posterior quadrant. This may introduce some bias as compared with whole‐head coverage. An interesting comparison would have been to directly compare the OP‐MEG recordings with those made using cryogenic MEG. This was not possible in this case due to the patient’s surgical schedule and ethical constraints. However, based on previous empirical studies,^8^, ^14^ we would have expected a twofold to fivefold reduction in signal magnitude using conventional MEG. The advantages of conventional MEG in this case would have been increased channel number and whole‐head coverage. We are currently working toward a system using 50 OPM devices where we should expect to see comparable whole‐brain source‐level discrimination^15^ to a cryogenic system with approximately 250 sensors. Further development of the technique will include simultaneous 30‐channel OP‐MEG and EEG recording, which has already been demonstrated in healthy subjects^16^ and prolonged EEG/OP‐MEG recording over several hours with video monitoring. Work is also underway in developing OP‐MEG for children with refractory epilepsy where motion tolerance is of even more importance. In addition, a new large OP‐MEG recording suite (3 x 4 meters) has been completed at Wellcome Centre for Human Neuroimaging, UCL, with improved low‐frequency shielding to allow a larger area for the patient to move in while recording. OP‐MEG has the potential to improve the quality of functional imaging within epilepsy diagnostics and finally translate MEG into a readily clinically available tool.

## Conflict of Interest

This work was in part funded by a Wellcome collaborative award, which involves a collaborative agreement with the OPM manufacturer QuSpin. The design for biplanar magnetic field compensation coils, as used in this study, has been patented by the University of Nottingham.

## Supporting information


**Figure S1** **.** A. Example traces of typical epileptiform activity from scalp EEG and OP‐MEG from first recording session; including spike and wave, polyspike and wave, and sharp wave.
