# Supplementary figures and images for: Optically pumped magnetoencephalography in epilepsy
Source: Ann Clin Transl Neurol. 2020 Feb 29;7(3):397–401. doi: 10.1002/acn3.50995 (PMC7085997; doi:10.1002/acn3.50995)

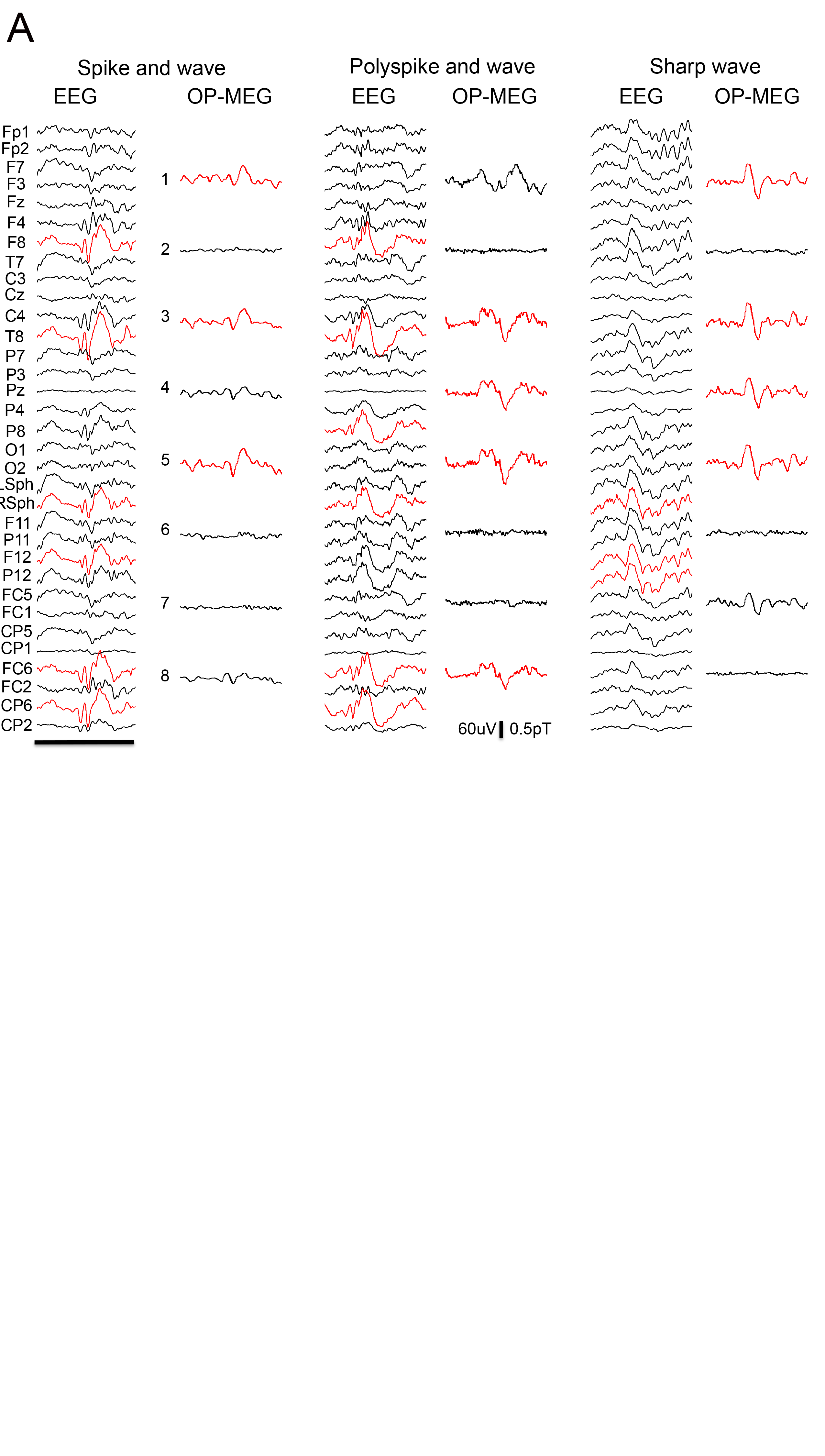

Supplement: Supplementary file 1 — Figure S1 . A. Example traces of typical epileptiform activity from scalp EEG and OP‐MEG from first recording session; including spike and wave, polyspike and wave, and sharp wave. [file ACN3-7-397-s001.tif]
